# A comparison of three post-activation potentiation enhancement warm up strategies on bench press performance

**DOI:** 10.3389/fspor.2025.1711615

**Published:** 2025-12-18

**Authors:** Yousef Hedayati, Roland van den Tillaar

**Affiliations:** 1 Department of Sports Biomechanics, Faculty of Physical Education and Sports Science, Shahid Bahonar University of Kerman, Kerman, Iran; 2 Department of Sport Sciences and Physical Education, Nord University, Levanger, Norway

**Keywords:** warming-up, resistance training, strength, post-activation potentiation (PAP), post-activation performance enhancement (PAPE)

## Abstract

**Background:**

At loads greater than 80% of one repetition maximum in bench press, at the ascending phase of the movement, the barbell decelerates or stops for a short time before it accelerates again during a region named sticking region. Post-Activation Performance Enhancement is one of the ways to increase maximal performance in bench press.

**Objective:**

The present study was conducted with the aim of investigating the effects of different warm-up protocols with ballistic, high resistance exercises or dynamic stretching on the maximal barbell bench press performance and elbow angular velocity around the sticking region in male students.

**Methods:**

Eighteen resistance trained male students (age: 23.8 ± 1.3 years, height: 1.70 ± 0.04 m, body mass: 74.4 ± 3.8 kg) performed maximal bench press after three different warm-up strategies, while barbell velocity, elbow angular velocity and barbell displacement around the sticking region were analysed with a 3D motion analysis system.

**Results:**

The barbell velocity after the ballistic warm-up at first maximal velocity from the lowest position on the chest (v_max1_), lowest barbell velocity after maximal velocity (v_min_) and second maximal barbell velocity (v_max2)_ were higher compared to the other two warm-up methods. As well as, the time of occurrence of v_min_ and v_max2_ occurred significantly earlier at the ballistic warm-up compared to the other two warm-ups, while the barbell height at v_max1_ after the ballistic warm-up was significantly higher than after the other two warm-up protocols. Furthermore, elbow angular velocity was higher after the ballistic warm-up protocol compared to the other two protocols with significant differences observed at v_max1_ and v_min_ and not at v_max2_.

**Conclusion:**

According to the obtained results, warm-up with ballistic exercises could have more optimal effects on athletes' performance and resulted in better surpassing the sticking region of the barbell bench press.

## 1 Introduction

Bench press is one of the most popular lifts used in strength training for the upper body. The lift is typically performed lying supine on a bench, using a barbell in which the barbell is first lowered to the chest and then pushed up until the elbows are fully extended (1).

A number of chronic and acute techniques have been suggested to enhance neuromuscular performance. Evans (2) proposed that chronic neuromuscu­lar augmentations are related to a wide range of muscular strengthening strategies and periodization approaches. Regarding acute performance enhancements, it has been speculated that performance improvement may be obtained through warm-up techniques. The utilization of low volume and moderate or high intensity conditioning contractions is considered as one of the most useful strategies, causing significant neuromuscular improvements in the primary mover muscles (3).

Blazevich and Babault (3) found that through a phenomenon called post-activation potentiation (PAP), these performance improvements occur less than three minutes after implementing an intensive vol­untary muscular contraction. However, post-activation performance enhancement (PAPE) with the aim of enhancing following voluntary force production, occurs after an applied appropriate rest period (7–10 min after the conditioning contraction) following a high-intensity exercise warm-up (4).

There are several exercise factors, determining PAPE to enhance following performance, such as load and type of exercise (5, 6). Timon, Allemano (5) suggested that either low-load (30%–40% of 1-RM) ballistic exercise or high resistance-load (85% of 1-RM) traditional exercises would be the most effective resistance exercises. The ballistic exercises are known to activate fast-twitch type II muscle fibers due to the recruitment of higher order motor units (7). Thereby, Ulrich and Parstorfer (7) suggested that an explosive-based movement can elicit an increased amount of motor neuron excitability and an enhanced motor unit recruitment pattern, which may have led to an augmentation of PAPE and result in enhancement of performance. On the other hand, the use of high resistance-load as priming is based upon the idea that a conditioning stimulus, which is similar to the actual performance, causes increasing the specificity of the conditioning stimulus activity (8). The specificity is defined as the level of bioenergetics and biomechanical similarity between training modes and approaches with performance (9). Traditional warm-up before 1-RM bench press with heavy resistance exercises might be used to simulate the actual movement with submaximal and near maximal loads.

Furthermore, performing dynamic stretching, before sports skills, by increasing the range of motion, body temperature and blood flow in active muscles is also suggested to improve the performance of the athlete (10). These exercises, which simulate the movement pattern used in a sport activity, may increase coordination by providing an opportunity to practice a specific sport skill (11, 12).

It has been suggested that in successful or unsuccessful bench press, at loads greater than 85% of 1-RM (13, 14), there is a region called the “sticking region” during the concentric phase of the movement at which the barbell decelerates or completely stops for a short moment before it starts accelerating again. In this region most lifts result in failure, thereby surpassing this region increases the possibility to succeed (13, 14). However, to our knowledge, limited research has been conducted on the effect of PAPE warm up with ballistic, high resistance exercises or dynamic stretching in 1-RM bench press. Therefore, the aim of the present study is to investigate the effect of PAPE warm up using ballistic, high resistance exercises and dynamic stretching on bench press performance around sticking region and elbow angular velocity. It is hypothesized that a higher barbell velocity and elbow angular velocity would occur around the sticking region after performing ballistic warm-up strategy due to the greater power output development and optimal induced PAPE (10) compared to high resistance exercises or dynamic stretching prior to the 1-RM, while a heavy resistance exercises protocol would also be more effective on athlete's performance around the sticking region compare to the dynamic stretching of prime movers in bench press due to the specific principle (8).

## 2 Materials and methods

**Figure 1:**
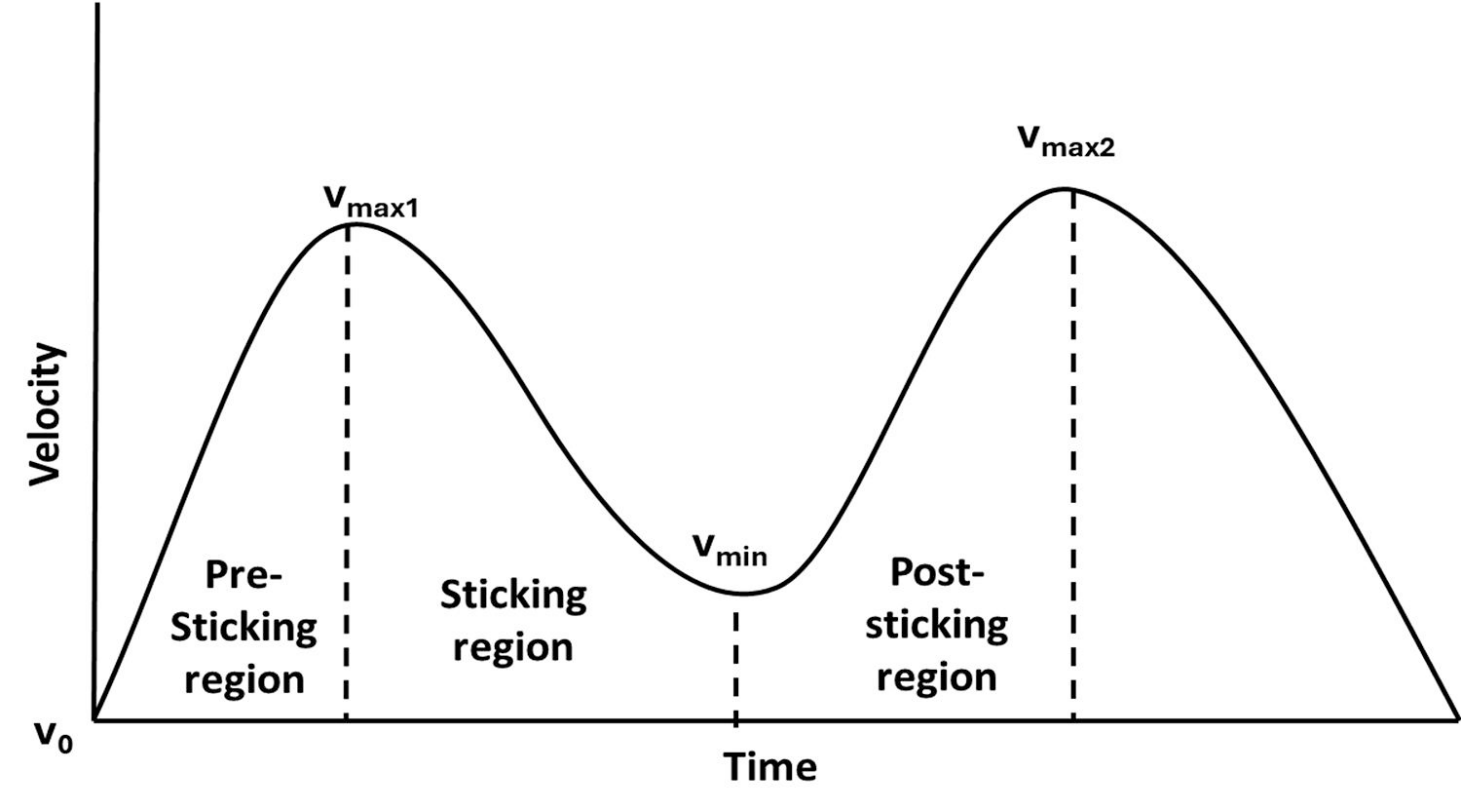
Barbell velocity development during 1-RM in a bench press exercise with the different events and regions.

This study assessed the effect of three different warm-up strategies upon maximal 1-RM bench press performance around the sticking region included pre-sticking, sticking and post-sticking regions. These regions are based upon the barbell velocity as the primary indicator of passing the sticking region as from the lowest barbell point (v_0_) velocity increases until first peak barbell velocity (v_max1_: pre-sticking region), followed by a decrease in velocity the first located lowest vertical velocity (v_min_: sticking region), after which the barbell velocity again increases until the second peak velocity (v_max2_: post-sticking region) (Figure 1). The sticking region is also identified as the region in which lifting attempts most often do not succeed (15). By using these regions this will give more information about the effects of the different warm-up strategies. In addition, angular elbow extension at the different events (v_max1_, v_min_ and v_max2_) was identified as this joint movement is responsible for the barbell velocity. Therefore, these variables as the resultant of neuromuscular performance affected by three warm-up strategies to overcome the sticking region were analysed. The independent variables were the ballistic exercises, heavy resistance exercises and dynamic stretching warm-up protocols. Dependent variables were the barbell velocity, elbow angular velocity and the barbell distance during the ascending phase of the bench press and arounding the pre-sticking, sticking and post-sticking regions.

### 2.1 Participants

Eighteen physical education students [age: (23.8 ± 1.3 years), height: (1.7 ± 0.04 m), body mass: (74.4 ± 3.8 kg)] with at least 1 year of resistance training experience volunteered in this study. All participants were familiar with the bench press exercise. The participants were instructed to avoid doing any additional resistance training for upper body during the 48 h before testing and a written consent was obtained prior to the study from all of them. Ethics approval was obtained from the local research ethics committee of Tehran university. (Ethics id: IR.UT.SPORT.REC.1402.136) and following the latest version of the Declaration of Helsinki.

### 2.2 Procedures

One week prior to the experimental test, 1-RM was measured for all participants according to Wilk, Golas (16). The participants were lying supine with a flexion of approximately 90° in the knees. They performed a standardized general and specific warm-up before testing. The warm-up protocol included a 5 min cycling on a stationary ergometer (heart rate of around 130 bpm), followed by a general upper-body warm-up of 10 trunk rotations and trunk side-bends on each side, 10 internal and external rotary movements of the shoulders, and 10 push-ups. Next, the participants performed 15, 10, and 5 repetitions using 20%, 40%, and 60% of their estimated 1-RM with a medium grip width (17). The first test load was set to an estimated 80% 1-RM and was increased by 2.5–10 kg for each subsequent attempt. This process was repeated until failure. During the 1-RM test, 5 min rest intervals were given between each attempt and the 1-RM was attained within 5 attempts. Each participant selected a medium grip and feet position, which were controlled for during the rest of the study. The participants were assisted by two spotters in the preload phase by lifting and stabling the Olympic barbell (2.8 cm diameter, length 1.92 m) until the participant had fully extended arms. In a controlled manner the barbell was lowered until it lightly touched the chest and was lifted back to the starting position with fully extended elbows. No bouncing of the weight was allowed. The time of each repetition was self-selected, but the participants were instructed to use a controlled tempo with correct technique and with maximal effort (14).

On test day the participants started with a 10 min' general warm-up, which included 4 min cycling on a stationary cycle ergometer (<100 w at 60–80 rpm), 3 min walking with shoulder rotation, 10 Lunges with torso rotation and 10 high knees. After this they performed one of the three specific warm-up protocols (Table 1). The three warm-up protocols were (1) dynamic stretching of the primary movers involved in bench pressing, such as pectoralis major, anterior deltoid, triceps brachii, and biceps brachii muscles (18), (2) high resistance exercise and (3) ballistic exercise: a combination of resistance loads and elastic band: CX mega fitness MF107 (Mega Fitness, Attica, Greece). To perform ballistic exercises with elastic band, the participants lay down on a bench and held the handles of the elastic band that was fixed to the bench. The elastic band gave a resistance of ≈30 kg at the top of the bench press lift. The participants were asked to perform bench press with elastic band as fast as possible during one-minute period to simulate barbell bench press throw. After each warm-up protocol the participants had eight minutes rest before performing the test.

**Table 1:** Warm-up protocols.

Protocol	Exercise	Repetition/Duration	Intensity	Between set rest
Dynamic stretching	Dynamic stretching of the pectoralis major Dynamic stretching of the anterior deltoid Dynamic stretching of the triceps	5 min	Moderate	1 min
		5 min		
		5 min		1 min
High resistance exercise	Free-Weight bench press	10 Repetitions	20% 1-RM	1 min
		6 Repetitions	40% 1-RM	1 min
		6 Repetitions	60% 1-RM	2 min
		3 Repetitions	75% 1-RM	3 min
		2 Repetitions	85% 1-RM	5 min
Ballistic exercise	Free-Weight bench press Bench press with elastic band	10 Repetitions	20% 1-RM	2 min
		6 Repetitions	40% 1-RM	1 min
		3*1 min	Moderate	1 min
	Rest before actual test			8 min

The participants were randomly divided into three groups (A–C) to ensure different order of testing the three warm-up protocols (Amiri-Khorasani, 2015) and were performed with 48 h' rest in between, to avoid the effect of fatigue on the results of each session (Table 2).

**Table 2:** Order of warm-up protocols of the different groups for testing at different days.

Test day	1	2	3
Group A	Ballistic exercise	High resistance exercise	Dynamic stretching
Group B	Dynamic stretching	Ballistic exercise	High resistance exercise
Group C	High resistance exercise	Dynamic stretching	Ballistic exercise

### 2.3 Measurements

**Figure 2:**
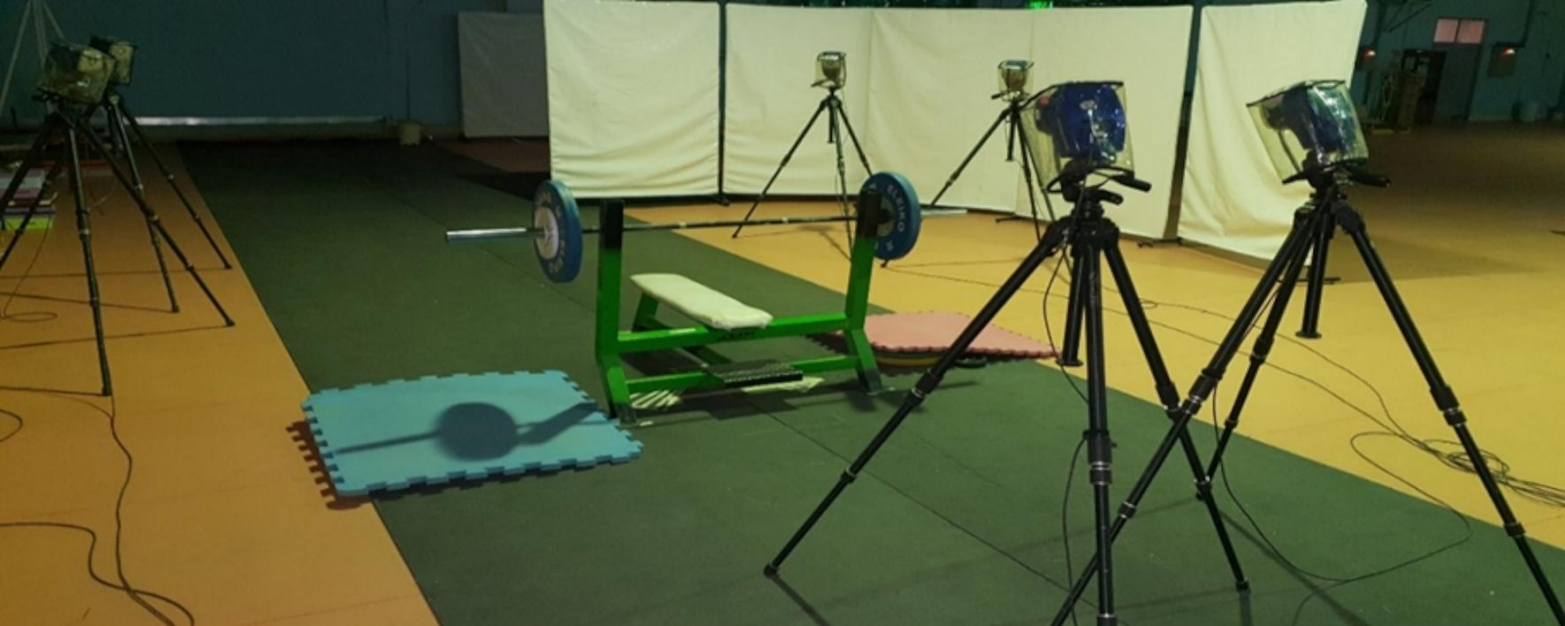
Experimental space with camera positions and bench press table.

A three-dimensional motion capture system (Digital Real Time System, Raptor-H, 200 Hz) with six cameras was utilized to record kinematics of the upper extremity. The cameras were placed around the bench press (Figure 2) and the experimental space was calibrated by a L-frame for static calibration and a T-vand (500 mm length) for dynamic calibration. participants attended the biomechanics lab. After executing their warm-up protocols according to the Tables 1, 2, three reflective markers (1.9 cm diameter) were put on the anatomical landmarks on each side of participants' body as follows: lateral tip of the acromion, lateral epicondyle of the elbow and the styloid process of the ulna. Each marker was identified by at least two cameras to analyze three-dimensional kinematics of the elbow. Moreover, as barbell velocity was calculated by using two markers attached to the middle of the barbell (0.2 m from each other) for identification of the three regions: pre-, sticking and post-sticking (Figure 1) during one attempt including the eccentric and concentric phases of the movement (17). In test days A–C, each 1-RM kinematics measurement was performed once for each participant with the load was identified in the 1-RM testing session. Elbow flexion/extension angles were determined from lines formed between the centers of the reflective markers on the acromiom, elbow and ulna for the whole attempt. Signals were bandpass filtered (50–400 Hz), rectified and integrated with 100 ms width.

To measure barbell velocity, the average vertical displacement of two markers attached to the middle of the barbell was used and the different events around the sticking region (v_0_, v_max1_, v_min_ and v_max2_) were identified. To compare elbow angular velocity during the ascending phase of the exercise, the average angular elbow extension velocity during the pre-, sticking and post-sticking regions was calculated.

### 2.4 Statistical analysis

All raw data were transformed into MATLAB software versin 2022a and Statistical analysis was performed in SPSS version 25.0. The Shapiro–Wilk test was used to verify the data's normality. A one-way ANOVA with repeated measures with Holm-Bonferroni *post hoc* testing was used to identify differences in kinematics between the barbell velocity, elbow angular velocity and barbell displacement for three warm-up protocols, considering a *p* < 0.05 value as statistically significant. Effect size was evaluated with *η*^2^ (ETA squared), where 0.01 < *η*^2^ < 0.06 constitutes a small effect, 0.06 < *η*^2^ < 0.14 constitutes a medium effect, and *η*^2^ > 0.14 constitutes a large effect (19).

## 3 Results

**Figure 3:**
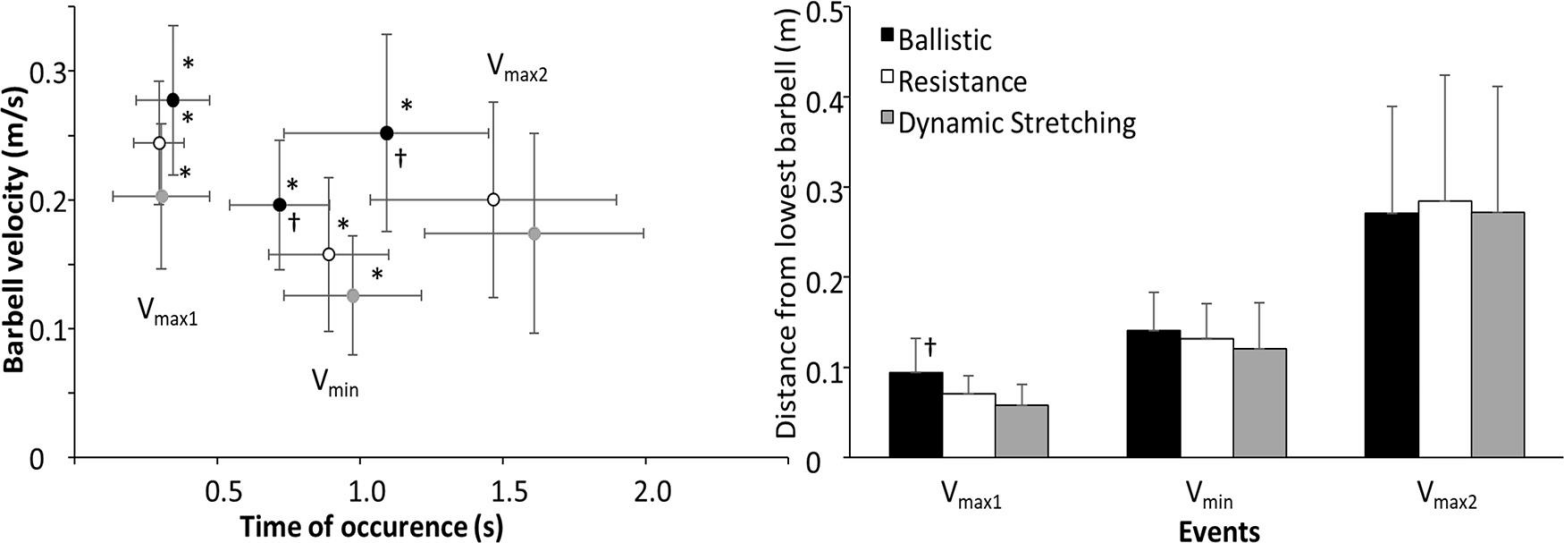
Mean (±SD) barbell velocity and displacement at the different events after the three warm-up protocols. * indicates a signficant difference in velocity with all other protocols for this event (*p* < 0.05). ^†^ indicates a signficant difference in time of occurrence and distance with all other protocols for this event (*p* < 0.05).

A signficant effect of the different warm-up protocols was found for the barbell velocity at all three events around the sticking region (*F* ≥ 10.1, *p* < 0.001, *η*^2^ ≥ 0.37), while for the time of occurrence of the different events only a signficant effect was found at v_min_ and v_max2_ (*F* ≥ 9.7, *p* < 0.001, *η*^2^ ≥ 0.36). The distance that the barbell had travelled at the different events was only significantly effected at v_max1_ (*F* = 11.8, *p* < 0.001, *η*^2^ = 0.41). *Post hoc* comparison revealed that the barbell velocities after the ballistic warm-up in all events were higher than compared with the other two warm-up protocols. Also at v_max1_ and v_min_ the barbell velocity after the heavy resistance warm-up was signficantly higher than after dynamic stretching. The time of occurrence of v_min_ and v_max2_ occurred signficantly earlier after the ballistic warm-up compared to the other two warm-ups, while the barbell height at v_max1_ after the ballistic warm-up was significantly higher than after the other two warm-up protocols (Figure 3).

**Figure 4:**
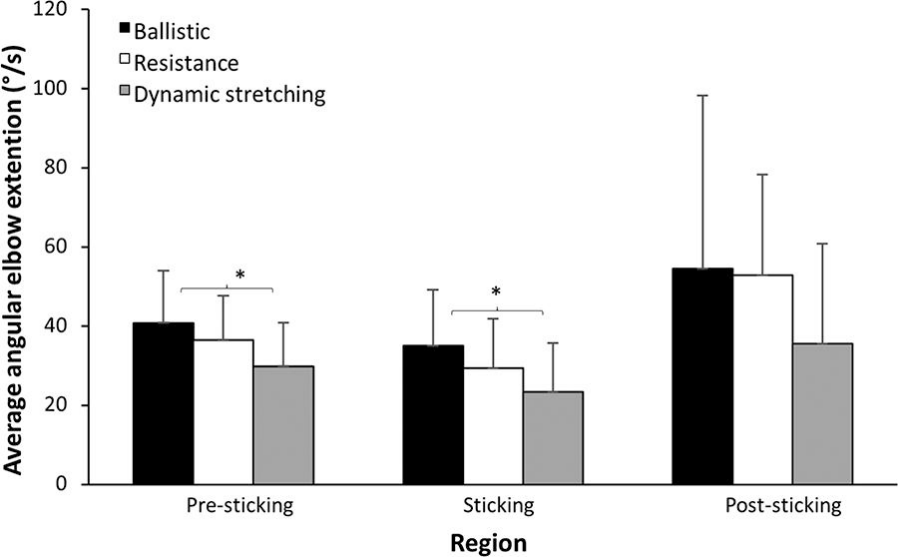
Mean (±SD) angular elbow extension at the different events after the three warm-up protocols. * indicates a signficant difference in velocity with between these two protocols for this event (*p* < 0.05).

The angular velocity of elbow extension was also affected at the different events. A signficant effect was observed at v_max1_ and at v_min_ (*F* ≥ 4.9, *p* ≤ 0.013, *η*^2^ ≥ 0.22) and not at v_max2_ (*F* = 2.58, *p* = 0.091, *η*^2^ = 0.13). *Post hoc* comparison revealed that angular velocity was higher after the ballistic warm-up protocol compared to the dynamic protocol at v_max1_ and v_min_ (Figure 4).

## 4 Discussion

The aim of this study was to compare the effects of different warm-up protocols with ballistic, high resistance exercises or dynamic stretching on the maximal barbell bench press performance and elbow angular velocity around the sticking region. The main findings were that barbell velocity was significantly higher at all measured points around the sticking region and all these events occurred earlier after the ballistic warm-up compared to the other two warm-ups (Figure 3). This higher barbell velocity was accompanied by an overall higher elbow extension velocity at both v_max1_ and v_min_ after the ballistic warm-up (Figure 4). In addition, the barbell velocity at v_max1_ and v_min_, following the heavy resistance warm-up was higher than after the dynamic stretching warm-up (Figure 3), which supporting both of our hypotheses.

Velocities at the different events were comparable to those reported in earlier studies on maximal bench press (14, 15). After the ballistic warm-up, the barbell traveled a greater distance upward from v_0_ to v_max1_ compared to the other two protocols. This increased displacement was due to the barbell's higher velocity at v_max1_ (Figure 3), which was in turn due to the higher angular elbow velocity (Figure 4). Additionally, higher barbell velocities at v_min_ and v_max2_ were observed following the ballistic warm-up compared to the other two protocols, indicating that this warm-up had a greater positive effect on bench press performance as it allowed by enabling faster movement through the sticking region, which enhances the possibility to surpass this region in which most failures in bench press occur. These increased barbell velocities were likely the result of faster joint movements, as evidenced by higher elbow extension velocity (Figure 4).

These findings align with previous studies (20, 21), which reported a positive increase in barbell velocity after performing ballistic barbell bench press movements as a pre-activation stimulus before the 1-RM bench press. Only Bodden, Suchomel (22) found that both ballistic and non-ballistic bench press conditioning stimuli decreased subsequent performance. The difference between this study and present study is related to lack of an adequate neuromuscular enhancement caused by ballistic conditioning contraction compared to non-ballistic one for improving subsequent exercise. However, the discrepancy between our study and Bodden, Suchomel (22) may be explained by differences in protocol. Bodden, Suchomel (22) used 13 repetitions at various percentages of 1-RM (30%–90%) as the conditioning stimulus, followed by only one minute of rest before testing plyometric push-up performance. Although push-ups and bench press share similar movement patterns (23), performing repetitive plyometric push-ups after only one minute of rest is likely more affected by fatigue than maximal bench press after an eight minute ballistic warm-up, as in the present study.

This enhancement of performance is probably caused by post-activation performance enhancement (PAPE) (24), which, as suggested by Blazevich and Babault (3), becomes significant only after several minutes. There are several mechanisms behind PAPE and optimized bench press performance following ballistic warm-up strategy. The ballistic warm-up, involving rapid downward and upward bench press movements, could stimulate the stretch-shortening cycle with minimal amortization time during the transition from eccentric to concentric muscle contractions, thereby effectively utilizing stored elastic energy (25). Furthermore, ballistic warm-up may enhance potentiation of contractile elements and increase activation of the pectoral and deltoid muscles, the prime movers in the bench press (15).

Another possible mechanism is that during ballistic contractions, the threshold for motor unit recruitment is lower than during slower ramped contractions (26, 27). This reduction in recruitment threshold may be the primary reason ballistic exercises provide an effective stimulus for PAPE. The strong excitatory drive during ballistic contraction enables activation of the entire motor neuron pool within a few milliseconds (28). While the recruitment threshold is lower during ballistic contractions than ramped contractions, there does not appear to be selective recruitment of faster motor units, and the size principle of contraction is largely preserved (26, 27, 29). However, no electromyographic measurements were performed in the present study to support these statements.

Moreover, our findings revealed that after heavy resistance exercises, barbell velocity was significantly higher at v_max1_ and v_min_ compared to dynamic stretching but lower than after the ballistic warm-up. This may be explained by greater fatigue of central and peripheral origin following heavy resistance loading, which reduces muscle electrical activity and is accompanied by blood lactate accumulation, compared to explosive and lighter loading (30). Additionally, using lower external loads may reduce exercise-induced muscle damage (31). Based on these observations, ballistic exercises appear to be an appropriate priming strategy for minimizing negative factors that impair subsequent neuromuscular performance.

Although several studies have suggested that dynamic stretching is the optimal stretching technique during warm-up to elicit PAPE (32–34), by increasing range of motion, body temperature, and blood flow in active muscles to improve athletic performance (10), our study indicated that dynamic stretching warm-up was less effective than ballistic or resistance-based warm-up in enhancing subsequent bench press performance. This may be because dynamic stretching was not specific enough, as it did not simulate the bench press movement (35). Therefore, dynamic stretching did not enhance neuromuscular patterns and muscle-tendon unit readiness to the same extent as the other two warm-ups (3, 36).

Interestingly, faster velocities at the different events after the three warm-up protocols resulted only in a higher barbell height at v_max1_ following the ballistic warm-up; barbell heights at v_min_ and v_max2_ were similar across all three warm-ups. This was due to the shorter time of occurrence after the ballistic warm-up (Figure 3). This suggests that the end of the sticking region (v_min_, also called the sticking point) is primarily caused by a poor mechanical region (18), which must be overcome to increase the chance of a successful lift.

The present study has some limitations. Firstly, we analyzed only velocities and timing of events; muscle activity of prime movers was not evaluated using EMG. Secondly, the ballistic warm-up protocol was performed with an elastic band; future studies could explore the use of barbell bench press throws. Thirdly, the study examined only acute effects, with no longitudinal follow-up to determine if such improvements translate into long-term strength gains. Fourthly, we used recreationally trained subjects rather than experienced powerlifters. Studies involving professional powerlifters could confirm our findings in those populations.

## 5 Conclusion

It was concluded that ballistic exercise warm-up caused better lifting performance (PAPE effect) around the sticking region than the other two warm-up protocols. This better performance was caused by the higher average elbow extension at the pre-sticking and sticking region. Ballistic bench press warm-up, due to its effect on the reduction of amortization phase of the stretch shortening cycle and utilizing optimal elastic energy can potentiate the contractile elements and better activation of prime movers. All in all, the sticking region, as the most challenging part of the bench press at near maximal loads could be overcome by using ballistic exercises warm-up. These results give coaches and athletes (especially in powerlifting) a practical and simple equipment warm-up strategy before performing 1-RM and near 1-RM bench press.

## Data Availability

The raw data supporting the conclusions of this article will be made available by the authors, without undue reservation.
